# Differential diagnosis of disinhibited behaviors in forensic psychiatry: a report of three cases

**DOI:** 10.1192/j.eurpsy.2025.1523

**Published:** 2025-08-26

**Authors:** K. Şahin Akkaya, K. Çifteci, B. Dizman, O. Uyanık, E. Sönmez Güngör

**Affiliations:** 1 Psychiatry, Erenkoy Mental and Nervous Diseases Training and Research Hospital; 2 Neurology, University of Health Sciences, Sancaktepe Şehit Prof. Dr. İlhan Varank Training and Research Hospital, İSTANBUL, Türkiye

## Abstract

**Introduction:**

In forensic psychiatry, the most common diagnoses among the affected population are mood and psychotic disorders, personality disorders, or other conditions including intellectual disabilities. These individuals are either offenders or victims in criminal cases. Moreover, the tendency towards criminal behavior, disinhibition, agitation and aggression may also be associated with a neurocognitive disorder. Criminal behavior has been detected in 37.4% of frontotemporal dementia, 7.7% of Alzheimer’s disease, and 20% of Huntington’s disease patients.

**Objectives:**

Raising awareness on the potential presentations of disinhibited behaviors through three case reports and on the importance of detailed clinical evaluation to differentially diagnose and appropriately manage forensic referrals.

**Methods:**

Each case was evaluated based on a predefined procedure comprising the anamnesis, mental status examination, review of previous medical and forensic records, neurological consultation including neuropsychiatric battery, social investigation and any other relevant information. Informed consent was obtained from all patients before presentation.

**Results:**

Case 1

A 63-year-old male, presented with a legal request of determining the need for a legal representative. The patient, who previously showed no similar behavior, repeatedly took loans under his own name, sold his assets at undervalued prices, experienced difficulties managing money, and was easily deceived. The patient was determined to exhibit frontal-type memory impairment, consistent with behavioral variant FTD (bvFTD), and it was deemed appropriate to appoint him a legal representative.

Case 2

A 52-year-old male, presented to assess his criminal responsibility regarding a theft offense. Anamnesis revealed forgetfulness, difficulties in managing money, tendency to take items from stores without paying, and repetitive behavior of cooking. Detailed examination and tests conducted led to a diagnosis of anxiety disorder, with a conclusion indicating full criminal responsibility.

Case 3

A 72-year-old male was hospitalized regarding an insult towards legal authorities. During the observation period in the inpatient ward, he exhibited similar behaviors. However, the final diagnosis was consistent with no significant psychopathology, and the patient was deemed criminally fully responsible for this act.

**Conclusions:**

In cases where behavioral disturbances predominate, the possibility of frontal lobe dysfunction should be considered. Nevertheless, temporality relationships in symptomatology and the course of the disease, as well as where, when, and how the criminal act happened should also be taken into account. Cases which have similar initial presentations may finally lead to unrelated diagnoses. Systematic evaluations are necessary to accurately guide the legal authorities as well as planning treatment.

**Disclosure of Interest:**

None Declared

